# (*E*)-4-{[(Morpholin-4-yl)imino]­meth­yl}benzo­nitrile

**DOI:** 10.1107/S1600536814014020

**Published:** 2014-06-21

**Authors:** Zeliha Atioğlu, Mehmet Akkurt, Aliasghar Jarrahpour, Roghayeh Heiran, Namık Özdemir

**Affiliations:** a Ilke Education and Health Foundation, Cappadocia Vocational College, The Medical Imaging Techniques Program, 50420 Mustafapaşa, Ürgüp, Nevşehir, Turkey; b Department of Physics, Faculty of Sciences, Erciyes University, 38039 Kayseri, Turkey; c Department of Chemistry, College of Sciences, Shiraz University, 71454 Shiraz, Iran; d Department of Physics, Faculty of Arts and Sciences, Ondokuz Mayıs University, 55139 Samsun, Turkey

**Keywords:** crystal structure

## Abstract

In the title compound, C_12_H_13_N_3_O, the morpholine ring adopts a chair conformation and its mean plane is inclined to that of the benzene ring by 16.78 (12)°. The N—N=C—C bridge, which has an *E* conformation, has a torsion angle of 173.80 (19)°. In the crystal, mol­ecules stack along the *a* axis but there are no significant inter­molecular inter­actions present.

## Related literature   

For background to the importance of Schiff bases, see: Dhar & Taploo (1982 ▶); Zheng *et al.* (2009 ▶); Guzen *et al.* (2007 ▶); Asif (2014 ▶); Hisaindee *et al.* (2013 ▶). For a related structure, see: Akkurt *et al.* (2013 ▶).
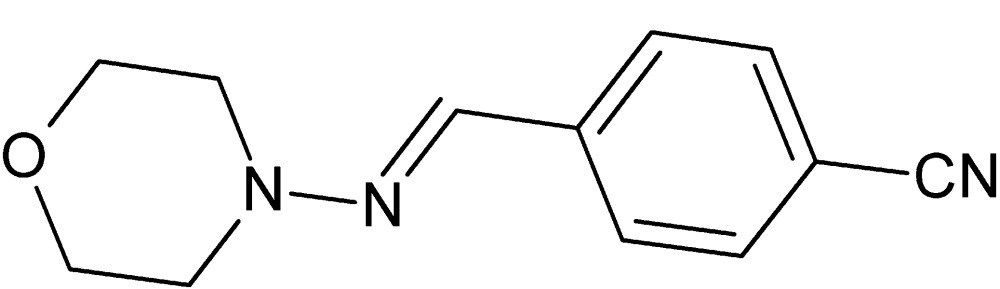


## Experimental   

### 

#### Crystal data  

C_12_H_13_N_3_O
*M* *_r_* = 215.25
Monoclinic, 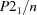

*a* = 4.1054 (3) Å
*b* = 12.0509 (8) Å
*c* = 22.9972 (19) Å
β = 91.087 (6)°
*V* = 1137.55 (15) Å^3^
*Z* = 4
Mo *K*α radiation
μ = 0.08 mm^−1^
*T* = 296 K
0.54 × 0.24 × 0.08 mm

#### Data collection  

Stoe IPDS 2 diffractometer
Absorption correction: integration (*X-RED32*; Stoe & Cie, 2002 ▶) *T*_min_ = 0.971, *T*_max_ = 0.994
6956 measured reflections
2394 independent reflections
878 reflections with *I* > 2σ(*I*)
*R*_int_ = 0.229

#### Refinement  

*R*[*F* ^2^ > 2σ(*F* ^2^)] = 0.063
*wR*(*F* ^2^) = 0.094
*S* = 0.89
2394 reflections
146 parameters
H-atom parameters constrained
Δρ_max_ = 0.12 e Å^−3^
Δρ_min_ = −0.11 e Å^−3^

### 

Data collection: *X-AREA* (Stoe & Cie, 2002 ▶); cell refinement: *X-AREA*; data reduction: *X-RED32* (Stoe & Cie, 2002 ▶); program(s) used to solve structure: *SHELXS2013* (Sheldrick, 2008 ▶); program(s) used to refine structure: *SHELXL2013* (Sheldrick, 2008 ▶); molecular graphics: *ORTEP-3 for Windows* (Farrugia, 2012 ▶); software used to prepare material for publication: *WinGX* (Farrugia, 2012 ▶).

## Supplementary Material

Crystal structure: contains datablock(s) global, I. DOI: 10.1107/S1600536814014020/su2744sup1.cif

Structure factors: contains datablock(s) I. DOI: 10.1107/S1600536814014020/su2744Isup2.hkl

Supporting information file. DOI: 10.1107/S1600536814014020/su2744Isup3.cml

CCDC reference: 1008300

Additional supporting information:  crystallographic information; 3D view; checkCIF report

## Figures and Tables

**Fig. 1.**
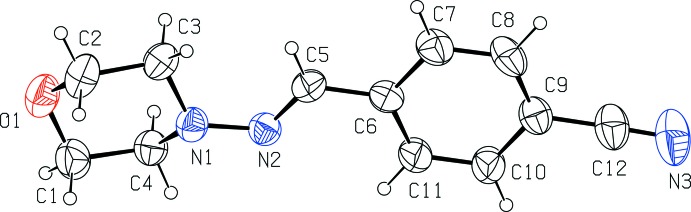
The molecular structure of the title molecule, with atom labelling. Displacement ellipsoids are drawn at the 30% probability level.

**Fig. 2.**
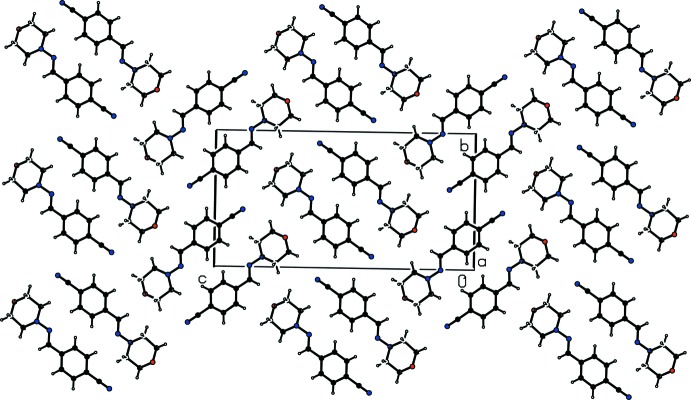
The crystal packing of the title compound, viewed along the a axis.

## supplementary crystallographic information

### S1. Comment

Schiff bases, also known as imines or azomethines, are some of the most widely used organic compounds, for example, as pigments and dyes, catalysts, polymer stabilizers, and as intermediates in organic synthesis in particular for the preparation of heterocycles (Dhar & Taploo 1982; Zheng *et al.*, 2009; Guzen *et al.*, 2007). Schiff bases also play an important role in biological systems with several applications, for example, as antifungal, anticancer, antibacterial, antimalarial, antiproliferative, anti-inflammatory and antiviral agents in addition to other biological performances (Asif, 2014; Hisaindee *et al.*, 2013). In view of this interest we synthesized the title compound and report herein on its crystal structure.

In the title molecule, Fig.1, the morpholine ring (N1/O1/C1–C4) adopts a chair conformation [puckering parameters: Q_T_ = 0.553 (3) Å, θ = 3.0 (3) ° and φ = 12 (6) °]. The N1–N2═C5–C6 torsion angle is is 173.80 (19) °. Bond lengths and angles are similar to those reported for a related structure (Akkurt *et al.*, 2013).

In the crystal structure, there are no classical hydrogen bonds. The crystal packing is stabilized by van der Waals interactions.

### S2. Experimental

The title compound was synthesized by refluxing 4-aminomorpholine (1.00 mmol) with 4-cyanobenzeldehyde (1.00 mmol) in ethanol for 30 min. It was then recrystallized from ethanol to give light-yellow prismatic crystals (Yield 86%; M.p.: 401–403 K). Spectroscopic data for the title compound are available in the archived CIF.

### S3. Refinement

All H atoms were placed in calculated positions and refined as riding: C—H = 0.93 and 0.97 Å for CH and CH_2_ H atoms, respectively, with *U*_iso_(H) = 1.2*U*_eq_(C). The crystals were of poor quality and weakly diffracting, which accounts for the low fraction of measured reflections. Repeated attempts to grow larger crystals were unsuccessful.

### Crystal data

**Table:**

C_12_H_13_N_3_O	*F*(000) = 456
*M**_r_* = 215.25	*D*_x_ = 1.257 Mg m^−^^3^
Monoclinic, *P*2_1_/*n*	Mo *K*α radiation, λ = 0.71073 Å
Hall symbol: -P 2yn	Cell parameters from 4198 reflections
*a* = 4.1054 (3) Å	θ = 1.7–27.1°
*b* = 12.0509 (8) Å	µ = 0.08 mm^−^^1^
*c* = 22.9972 (19) Å	*T* = 296 K
β = 91.087 (6)°	Prism, light yellow
*V* = 1137.55 (15) Å^3^	0.54 × 0.24 × 0.08 mm
*Z* = 4	

### Data collection

**Table:**

Stoe IPDS 2 diffractometer	2394 independent reflections
Radiation source: sealed X-ray tube, 12 x 0.4 mm long-fine focus	878 reflections with *I* > 2σ(*I*)
Plane graphite monochromator	*R*_int_ = 0.229
Detector resolution: 6.67 pixels mm^-1^	θ_max_ = 26.7°, θ_min_ = 1.8°
rotation method scans	*h* = −5→5
Absorption correction: integration (*X-RED32*; Stoe & Cie, 2002)	*k* = −13→15
*T*_min_ = 0.971, *T*_max_ = 0.994	*l* = −29→28
6956 measured reflections	

### Refinement

**Table:**

Refinement on *F*^2^	0 restraints
Least-squares matrix: full	Hydrogen site location: inferred from neighbouring sites
*R*[*F*^2^ > 2σ(*F*^2^)] = 0.063	H-atom parameters constrained
*wR*(*F*^2^) = 0.094	*w* = 1/[σ^2^(*F*_o_^2^) + (0.021*P*)^2^] where *P* = (*F*_o_^2^ + 2*F*_c_^2^)/3
*S* = 0.89	(Δ/σ)_max_ < 0.001
2394 reflections	Δρ_max_ = 0.12 e Å^−^^3^
146 parameters	Δρ_min_ = −0.11 e Å^−^^3^

### Special details

**Table:**

Experimental. Spectropscopic data for the title compound: IR (KBr, cm^-1^): 1604 (Schiff base C=N), 2214 (nitrile CN). ^1^H-NMR (250 MHz, CDCl_3_), δ (p.p.m.): 3.18–3.22 (CH_2_ morpholine ring, m, 4H), 3.83–3.87 (CH_2_ morpholine ring, m, 4H), 7.48 (HC=N, s, 1H), 7.54 (ArH, d, J=8.4 Hz, 2H), 7.62 (ArH, d, J=8.2 Hz, 2H). ^13^C-NMR (62.9 MHz, CDCl_3_), δ (p.p.m.): 51.3 (CH_2_N), 66.2 (CH_2_O), 110.7, 119.0, 126.2, 132.3, 132.6 (aromatic carbons and nitrile), 140.4 (C=N).
Geometry. Bond distances, angles *etc*. have been calculated using the rounded fractional coordinates. All su's are estimated from the variances of the (full) variance-covariance matrix. The cell e.s.d.'s are taken into account in the estimation of distances, angles and torsion angles
Refinement. Refinement on *F*^2^ for ALL reflections except those flagged by the user for potential systematic errors. Weighted *R*-factors *wR* and all goodnesses of fit *S* are based on *F*^2^, conventional *R*-factors *R* are based on *F*, with *F* set to zero for negative *F*^2^. The observed criterion of *F*^2^ > σ(*F*^2^) is used only for calculating -*R*-factor-obs *etc*. and is not relevant to the choice of reflections for refinement. *R*-factors based on *F*^2^ are statistically about twice as large as those based on *F*, and *R*-factors based on ALL data will be even larger.

### Fractional atomic coordinates and isotropic or equivalent isotropic displacement parameters (Å^2^)

**Table:**

	*x*	*y*	*z*	*U*_iso_*/*U*_eq_	
O1	0.3333 (4)	0.7856 (2)	0.26705 (10)	0.0934 (9)	
N1	0.5804 (4)	0.92897 (19)	0.18135 (10)	0.0661 (9)	
N2	0.6187 (4)	0.9921 (2)	0.13245 (9)	0.0642 (9)	
N3	0.9567 (8)	1.4147 (2)	−0.10077 (15)	0.1297 (14)	
C1	0.4673 (6)	0.8185 (2)	0.16642 (12)	0.0744 (11)	
C2	0.5031 (6)	0.7434 (3)	0.21844 (15)	0.0919 (15)	
C3	0.4574 (7)	0.8916 (3)	0.28163 (13)	0.0920 (14)	
C4	0.4229 (6)	0.9736 (2)	0.23245 (12)	0.0739 (11)	
C5	0.5801 (5)	1.0975 (3)	0.13421 (11)	0.0671 (10)	
C6	0.6545 (6)	1.1639 (2)	0.08316 (12)	0.0621 (10)	
C7	0.5920 (6)	1.2770 (2)	0.08316 (13)	0.0797 (12)	
C8	0.6662 (7)	1.3426 (2)	0.03595 (15)	0.0865 (14)	
C9	0.8027 (6)	1.2960 (3)	−0.01231 (14)	0.0732 (11)	
C10	0.8635 (6)	1.1833 (2)	−0.01327 (14)	0.0864 (12)	
C11	0.7892 (6)	1.1192 (2)	0.03383 (13)	0.0817 (12)	
C12	0.8858 (8)	1.3627 (3)	−0.06134 (17)	0.0941 (16)	
H1A	0.59330	0.78940	0.13460	0.0890*	
H1B	0.24050	0.82150	0.15390	0.0890*	
H2A	0.41920	0.67040	0.20860	0.1100*	
H2B	0.73230	0.73570	0.22870	0.1100*	
H3A	0.68600	0.88470	0.29250	0.1100*	
H3B	0.34310	0.91970	0.31510	0.1100*	
H4A	0.19410	0.98700	0.22380	0.0890*	
H4B	0.52330	1.04350	0.24350	0.0890*	
H5	0.50530	1.13130	0.16780	0.0800*	
H7	0.49840	1.30930	0.11550	0.0950*	
H8	0.62370	1.41830	0.03690	0.1040*	
H10	0.95490	1.15100	−0.04590	0.1030*	
H11	0.83060	1.04340	0.03250	0.0980*	

### Atomic displacement parameters (Å^2^)

**Table:**

	*U*^11^	*U*^22^	*U*^33^	*U*^12^	*U*^13^	*U*^23^
O1	0.0852 (12)	0.1033 (19)	0.0918 (16)	−0.0208 (12)	0.0086 (12)	0.0200 (14)
N1	0.0643 (13)	0.0668 (18)	0.0673 (15)	−0.0065 (11)	0.0016 (12)	0.0045 (14)
N2	0.0627 (12)	0.0629 (17)	0.0669 (15)	−0.0007 (12)	0.0013 (11)	0.0014 (13)
N3	0.191 (3)	0.086 (2)	0.112 (2)	−0.031 (2)	−0.002 (2)	0.0243 (19)
C1	0.0715 (16)	0.065 (2)	0.087 (2)	−0.0054 (14)	0.0074 (14)	0.0055 (17)
C2	0.0829 (18)	0.083 (3)	0.110 (3)	−0.0068 (17)	0.0039 (18)	0.022 (2)
C3	0.0882 (19)	0.115 (3)	0.073 (2)	−0.023 (2)	0.0043 (16)	0.015 (2)
C4	0.0708 (16)	0.082 (2)	0.069 (2)	−0.0124 (15)	0.0029 (15)	−0.0037 (17)
C5	0.0664 (16)	0.075 (2)	0.0600 (18)	0.0006 (15)	0.0063 (13)	−0.0042 (16)
C6	0.0647 (15)	0.0553 (19)	0.066 (2)	0.0010 (14)	−0.0046 (14)	−0.0092 (16)
C7	0.104 (2)	0.062 (2)	0.073 (2)	0.0079 (17)	−0.0001 (17)	−0.0130 (17)
C8	0.119 (2)	0.049 (2)	0.091 (3)	0.0085 (17)	−0.013 (2)	−0.0018 (19)
C9	0.0852 (18)	0.059 (2)	0.075 (2)	−0.0054 (16)	−0.0061 (17)	0.0007 (18)
C10	0.118 (2)	0.064 (2)	0.078 (2)	0.0066 (18)	0.0245 (18)	0.0028 (18)
C11	0.112 (2)	0.053 (2)	0.081 (2)	0.0108 (16)	0.0230 (18)	−0.0010 (18)
C12	0.125 (3)	0.068 (2)	0.089 (3)	−0.0177 (19)	−0.004 (2)	0.007 (2)

### Geometric parameters (Å, º)

**Table:**

O1—C2	1.423 (4)	C9—C12	1.431 (5)
O1—C3	1.413 (4)	C10—C11	1.370 (4)
N1—N2	1.369 (3)	C1—H1A	0.9700
N1—C1	1.449 (3)	C1—H1B	0.9700
N1—C4	1.455 (3)	C2—H2A	0.9700
N2—C5	1.281 (4)	C2—H2B	0.9700
N3—C12	1.144 (5)	C3—H3A	0.9700
C1—C2	1.505 (4)	C3—H3B	0.9700
C3—C4	1.507 (4)	C4—H4A	0.9700
C5—C6	1.458 (4)	C4—H4B	0.9700
C6—C7	1.387 (3)	C5—H5	0.9300
C6—C11	1.381 (4)	C7—H7	0.9300
C7—C8	1.382 (4)	C8—H8	0.9300
C8—C9	1.373 (5)	C10—H10	0.9300
C9—C10	1.381 (4)	C11—H11	0.9300
			
C2—O1—C3	109.3 (2)	O1—C2—H2A	109.00
N2—N1—C1	110.9 (2)	O1—C2—H2B	109.00
N2—N1—C4	121.2 (2)	C1—C2—H2A	109.00
C1—N1—C4	112.68 (19)	C1—C2—H2B	109.00
N1—N2—C5	120.6 (2)	H2A—C2—H2B	108.00
N1—C1—C2	109.8 (2)	O1—C3—H3A	109.00
O1—C2—C1	111.6 (3)	O1—C3—H3B	109.00
O1—C3—C4	112.7 (2)	C4—C3—H3A	109.00
N1—C4—C3	109.1 (2)	C4—C3—H3B	109.00
N2—C5—C6	119.4 (2)	H3A—C3—H3B	108.00
C5—C6—C7	119.9 (3)	N1—C4—H4A	110.00
C5—C6—C11	122.7 (2)	N1—C4—H4B	110.00
C7—C6—C11	117.4 (2)	C3—C4—H4A	110.00
C6—C7—C8	121.3 (3)	C3—C4—H4B	110.00
C7—C8—C9	120.0 (3)	H4A—C4—H4B	108.00
C8—C9—C10	119.5 (3)	N2—C5—H5	120.00
C8—C9—C12	120.9 (3)	C6—C5—H5	120.00
C10—C9—C12	119.6 (3)	C6—C7—H7	119.00
C9—C10—C11	119.9 (3)	C8—C7—H7	119.00
C6—C11—C10	121.9 (2)	C7—C8—H8	120.00
N3—C12—C9	178.8 (4)	C9—C8—H8	120.00
N1—C1—H1A	110.00	C9—C10—H10	120.00
N1—C1—H1B	110.00	C11—C10—H10	120.00
C2—C1—H1A	110.00	C6—C11—H11	119.00
C2—C1—H1B	110.00	C10—C11—H11	119.00
H1A—C1—H1B	108.00		
			
C2—O1—C3—C4	59.5 (3)	N2—C5—C6—C11	−5.0 (4)
C3—O1—C2—C1	−59.4 (3)	C5—C6—C7—C8	178.6 (2)
C4—N1—N2—C5	14.6 (3)	C11—C6—C7—C8	−0.9 (4)
C1—N1—N2—C5	150.2 (2)	C5—C6—C11—C10	−178.7 (2)
N2—N1—C1—C2	166.62 (18)	C7—C6—C11—C10	0.9 (4)
C4—N1—C1—C2	−53.9 (3)	C6—C7—C8—C9	0.4 (4)
N2—N1—C4—C3	−172.2 (2)	C7—C8—C9—C10	0.3 (4)
C1—N1—C4—C3	52.9 (3)	C7—C8—C9—C12	−179.1 (3)
N1—N2—C5—C6	173.80 (19)	C8—C9—C10—C11	−0.4 (4)
N1—C1—C2—O1	56.8 (3)	C12—C9—C10—C11	179.0 (3)
O1—C3—C4—N1	−55.9 (3)	C9—C10—C11—C6	−0.2 (4)
N2—C5—C6—C7	175.5 (2)		

## References

[bb1] Akkurt, M., Jarrahpour, A., Chermahini, M. M., Aberi, M. & Büyükgüngör, O. (2013). *Acta Cryst.* E**69**, o1571. 10.1107/S160053681302566X PMC3790431 24098250

[bb2] Asif, M. (2014). *J. Pharm. Pharm. Sci.* **1**, 1–10.

[bb3] Dhar, D. N. & Taploo, C. L. (1982). *J. Sci. Ind. Res.* **41**, 501–506.

[bb4] Farrugia, L. J. (2012). *J. Appl. Cryst.* **45**, 849–854.

[bb5] Guzen, K. P., Guarezemini, A. S., Órfão, A. T. G., Cella, R., Pereira, C. M. P. & Stefani, H. A. (2007). *Tetrahedron Lett.* **48**, 1845–1848.

[bb6] Hisaindee, S., Al-Kaabi, L., Ajeb, S., Torky, Y., Iratni, R., Saleh, N. & AbuQamar, S. F. (2013). *Arabian. J. Chem.* In the press. 10.1016/j.arabjc.2013.03.013.

[bb7] Sheldrick, G. M. (2008). *Acta Cryst.* A**64**, 112–122. 10.1107/S0108767307043930 18156677

[bb8] Stoe & Cie (2002). *X-AREA* and *X-RED32* Stoe & Cie, Darmstadt, Germany.

[bb9] Zheng, Y., Ma, K., Li, H., Li, J., He, J., Sun, X., Li, R. & Ma, J. (2009). *Catal. Lett.* **128**, 465–474.

